# Health-related quality of life and self-reported cognitive function in patients with delayed neurocognitive recovery after radical prostatectomy: a prospective follow-up study

**DOI:** 10.1186/s12955-021-01705-z

**Published:** 2021-02-25

**Authors:** Ursula Kahl, Sarah Callsen, Stefanie Beck, Hans Pinnschmidt, Franziska von Breunig, Alexander Haese, Markus Graefen, Christian Zöllner, Marlene Fischer

**Affiliations:** 1 Department of Anesthesiology, University Medical Center Hamburg-Eppendorf, Martinistrasse 52, 20246 Hamburg, Germany; 2 Institute of Medical Biometry and Epidemiology, University Medical Center Hamburg-Eppendorf, Hamburg, Germany; 3 Martini-Klinik, Prostate Cancer Center, University Medical Center Hamburg-Eppendorf, Hamburg, Germany; 4 Department of Intensive Care Medicine, University Medical Center Hamburg-Eppendorf, Hamburg, Germany

**Keywords:** Delayed neurocognitive recovery, Postoperative cognitive dysfunction, Quality of life, Cognitive failures, Radical prostatectomy, Prostate cancer

## Abstract

**Background:**

Delayed neurocognitive recovery (DNCR) is a common and serious complication after radical prostatectomy. We hypothesized that patients with DNCR in the early postoperative period would report reduced health-related quality of life (HRQoL) and more cognitive failures 12 months after surgery, compared with patients without DNCR.

**Methods:**

We performed a 12-month follow-up on 367 patients who had been enrolled in a prospective observational trial to study the incidence of DNCR after radical prostatectomy. Patients were screened for preoperative cognitive impairment and depression. We defined DNCR as a decline in cognitive function between days 3 and 5 after surgery, compared with baseline assessments. We evaluated HRQoL and cognitive failures 12 months after surgery with the 36-item Short Form Health Survey and the Cognitive Failures Questionnaire. General linear models were used to analyze associations of DNCR with HRQoL and cognitive failures.

**Results:**

Delayed neurocognitive recovery in the early postoperative period was significantly associated with self-reported cognitive failures (B for no DNCR =  − 0.411 [95% CI: − 0.798;0.024], *p* = 0.038), but not with physical (B = 0.082 [95% CI: − 0.021;0.186], *p* = 0.118) or mental HRQoL (B =  − 0.044 [95% CI: − 0.149;0.062], *p* = 0.417) 12 months after surgery. Preoperative depression screening scores were significantly associated with self-reported cognitive failures and both physical and mental HRQoL 12 months after surgery.

**Conclusions:**

Delayed neurocognitive recovery in the early period after radical prostatectomy has a long-term impact on patients’ daily lives by impairing memory, attention, action, and perception. Therefore, prevention of DNCR must be a priority for physicians and researchers. Consequent preoperative screening for depressive symptoms may facilitate early psycho-oncological intervention to improve postoperative HRQoL.

*Trials registration* DRKS00010014, date of registration: 21.03.2016, retrospectively registered.

## Background

Prostate cancer is the most common malignancy in men in Western countries [1]. Localized prostate cancer can be treated with a conservative or a surgical therapeutic approach [2]. As surgical techniques have advanced in recent years, radical prostatectomy, especially robot-assisted surgery, has become increasingly popular with patients and physicians In the majority of patients, radical prostatectomy is characterized by a low complication rate and a beneficial functional urological outcome [3]. Moreover, patients can expect to have excellent quality of life after radical prostatectomy [4].

However, delayed neurocognitive recovery (DNCR)—formerly postoperative cognitive dysfunction—is a common complication during the early postoperative period after radical prostatectomy [5]. Delayed neurocognitive recovery is defined as a postoperative decline in cognitive function affecting memory, information processing, and executive function [6]. It has been reported in up to 40% of patients at hospital discharge after non-cardiac surgery [7]. Evered and coworkers report that 43% of postoperative cognitive decline manifests seven days after cardiac surgery [8]. The prevalence of cognitive impairment is still around 12% three months after surgery [9]. Whether postoperative neurocognitive disorders persist beyond three months postoperatively is under debate. Some authors have reported cognitive function after 12 months to be similar to that of the general population, while other studies have not found a higher prevalence of cognitive impairment one year after non-cardiac surgery [10–12]. Delayed neurocognitive recovery is associated with premature exiting of the labor market, a higher dependency on social transfer payments, and increased morbidity and mortality [13]. However, little is known about the long-term impact of DNCR on health-related quality of life (HRQoL) and cognitive function. As the aim of modern medicine is not only to extend life expectancy but also to improve quality of life, exploring the long-term consequences of DNCR may be crucial for optimizing postoperative HRQoL and cognitive performance [14].

We hypothesized that patients who had been diagnosed with DNCR in the early postoperative period would report a lower HRQoL and more cognitive failures relating to perception, memory, and motor function 12 months after surgery, compared with patients without DNCR. Therefore, we followed up patients who had been enrolled in a prospective observational trial to study the incidence of DNCR after radical prostatectomy and to compare postoperative cognitive function in patients who had undergone robot-assisted and those who had undergone open retropubic radical prostatectomy.

## Methods

### Ethical information

Ethical approval for this study (protocol number PV4782) was obtained from the ethics committee at the Hamburg State Chamber of Physicians (chairperson Dipl.-Dok. Maike Habeck-Heyer) on August 26th, 2014. The study adheres to the ethical standards of the 1964 Declaration of Helsinki and its later amendments. All patients gave written informed consent prior to study participation.

### Aim, study design, setting, and participants

The aim of this study was to compare HRQoL and cognitive failures between patients with and without DNCR following radical prostatectomy for prostate cancer. Patients who had been enrolled in a prospective cohort study to assess the incidence of DNCR during the early postoperative period were eligible for study participation [5]. The study was performed at the Martini-Klinik, a high-volume prostate cancer center in Northern Germany. Details of the initial study protocol have been reported previously [5]. In brief, patients scheduled for radical prostatectomy were enrolled between January 2015 and March 2016. Requirements for study participation were age > 18 years, elective surgery, American Society of Anesthesiologists physical status classification I–IV, and excellent knowledge of the German language to perform the verbal components of the neuropsychological assessments. Patients were excluded from study participation if they had a history of central nervous system disease (e.g., ischemic stroke, transient ischemic attack, dementia, intracranial pathology, neurodegenerative disease).

Patients who had completed the pre- and postoperative neuropsychological assessments for diagnosis of DNCR were included in the current study and were evaluated for HRQoL and cognitive failures 12 months after radical prostatectomy. Eligible patients were contacted by post, phone, or email 12 months after surgery, accepting a delay of six months.

### Psychometric assessment

Neuropsychological assessment and the definition of DNCR have been described in detail previously [5]. Briefly, the Mini-Mental Status Examination and the Patient Health Questionnaire-9 were used to screen for pre-existing cognitive impairment or signs of depression. For the evaluation of pre- and postoperative cognitive function, we used a test battery consisting of the California Verbal Learning Test, the Trail Making Test, the Grooved Pegboard Test, and the Digit Span Forward task. Preoperative assessment was performed on the day of admission, and postoperative assessment was performed between 3 and 5 days after surgery. For each test, the difference between the pre- and postoperative results was calculated and divided by the baseline standard deviations to obtain a z-score. The sum of all z-scores was divided by the standard deviation of a sum of z-scores derived from normative data, resulting in a combined z-score [15, 16]. We defined DNCR as z-scores above 1.96 or below − 1.96 in at least two subcategories of the California Verbal Learning Test plus one other task or a combined z-score above 1.96 [17].

### Health-related quality of life and cognitive failures 12 months after surgery

Health-related quality of life describes “how well a person functions in their life and his or her perceived wellbeing in physical, mental, and social domains of health” [14]. For this study, we assessed HRQoL 12 months after surgery using the German version of the Short Form Health Survey (SF-36) [18]. It consists of 36 items covering eight health-related sections and results in a physical component sum score and a mental component sum score.

The term ‘cognitive failures’ refers to the patient’s experience of his cognitive inabilities in daily life such as failures in perception, memory, and motor function [19]. Cognitive failures 12 months after surgery were assessed with the Cognitive Failures Questionnaire (CFQ) [19]. The CFQ is a self-report measure that includes 25 questions about minor mistakes in everyday life, which are rated on a Likert scale from never (0) to very often (4).

### Surgery and anesthesia

Radical prostatectomy was performed using either a robot-assisted or open surgical approach. The choice of surgical technique was based on patient-related perioperative risk factors, oncological considerations, and patient preference. All patients received general anesthesia with either propofol-sufentanil or sevoflurane-sufentanil for anesthesia maintenance.

### Statistical analysis

Data are presented as median and interquartile range (IQR) or absolute numbers and percentages. For group comparisons, t-tests, Mann–Whitney U tests, chi-square tests, or Fisher’s exact tests were used as appropriate. To reduce the initial set of independent variables, we performed a categorical nonlinear principal components analysis. We used general linear models to analyze the association between DNCR in the early postoperative period and both HRQoL (physical and mental component scores) and cognitive failures 12 months after surgery. To achieve normally distributed data, all interval-scaled endpoints were transformed. Physical and mental component scores were transformed using the formula ln(70–SF36) prior to this analysis. We transformed the CFQ score by taking its square root. Variables that were selected based on categorical nonlinear principal components analysis were included in the initial multivariable model (see Additional file 1). Following a stepwise backward approach, we gradually reduced the initial model. Variables that caused a change in parameter estimates of > 10% or were statistically significant at a 0.05 level remained in the model.

SPSS Statistics 24 (IBM Deutschland GmbH) was used for statistical analyses. Figures were designed with SPSS 24 or GraphPad Prism 8 for Mac (GraphPad Software, San Diego, CA).

## Results

### Study population

**Fig. 1:**
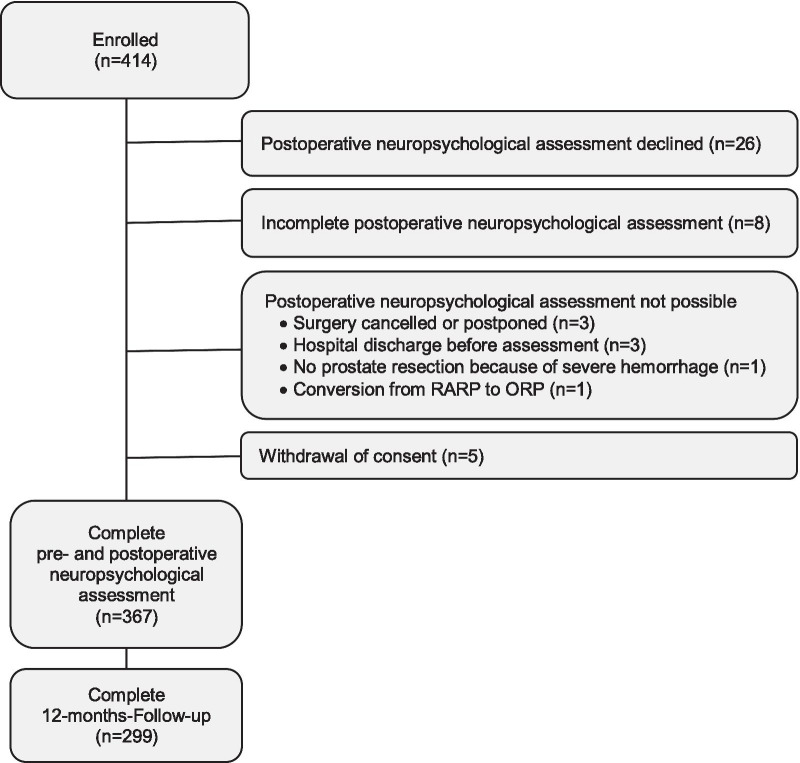
Flow diagram of study participants

Three hundred and sixty-seven patients with complete pre- and postoperative neuropsychological assessments were eligible for participation in the long-term follow-up. Of these, 299 patients (81.5%) completed the follow-up assessment of HRQoL and cognitive failures 12 months after surgery. The flow of participants throughout the study is presented in Fig. 1. The median age of the study population was 65 years (range 44–76 years). The majority of patients (n = 150 [83.6%]) had low perioperative risk and fulfilled the criteria for category I or II of the American Society of Anesthesiologists physical status classification. The median score on the Mini-Mental Status Examination was 29 (IQR: 28–30). More baseline demographic and clinical characteristics of the study population, stratified by the presence of DNCR, are summarized in Table 1.

**Table 1:** Baseline demographic and clinical characteristics

	no DNCR (n = 235)	DNCR (n = 64)
Age, years	64 (59–69)	66 (63–70)
Body Mass Index	25.8 (24.4–28.7)	25.7 (24.0–28.4)
*Education*		
< Highschool	100 (42.6)	27 (422)
≥ Highschool	131 (55.7)	36 (56.3)
Not reported	− 4 (1.7)	− 1 (1.6)
*ASA*		
I	52 (22.1)	8 (12.5)
II	145 (61.7)	45 (70.3)
III	38 (16.2)	11 (17.2)
Arterial hypertension	116 (49.4)	34 (53.1)
Coronary heart disease	22 (9.4)	5 (7.8)
Diabetes*	15 (6.4)	2 (3.1)
Dyslipoproteinemia	52 (22.1)	17 (26.6)
OSAS	12 (5.1)	2 (3.1)
COPD	8 (3.4)	2 (3.1)
Current smoking status	23 (9.8)	2 (3.1)
MMSE	29 (28–30)	29 (28–30)
PHQ-9	3 (2–5)	3 (2–5)

Data are given as absolute and relative numbers or median with interquartile range

*DNCR* delayed neurocognitive recovery, *ASA* American Society of Anesthesiologists physical classification system, *OSAS* obstructive sleep apnea syndrome, *COPD* chronic obstructive pulmonary disease, *MMSE* Mini-mental status examination (preoperative score), Patient Health Questionnaire 9 (preoperative score)

^*^Including Pre-diabetes

Of the 299 patients who were available for follow-up after 12 months, 64 had been diagnosed with DNCR in the early postoperative period [5]. Details of the neuropsychological test results are listed in Additional file 2. Robot-assisted radical prostatectomy was performed in 137 patients (45.8%), and 162 patients (54.2%) underwent open surgery. Variables related to surgery and anesthesia and uro-oncological features are presented in Table 2.

**Table 2:** Variables related to anesthesia and surgery and uro-oncological features, presented as numbers (%) or median with interquartile range

	no DNCR (n = 235)	DNCR (n = 64)
*Surgical technique*		
RARP	107 (45.5)	30 (46.9)
ORP	128 (54.5)	34 (53.1)
Duration of surgery, min	185 (160–210)	193 (163–218)
Estimated blood loss, ml	600 (300–900)	500 (250–900)
Midazolam for premedication	212 (91.4)	61 (95.3)
*Anesthetic technique*		
Balanced anesthesia	130 (55.3)	31 (48.4)
TIVA	105 (44.7)	33 (51.6)
Additional spinal anesthesia	60 (20.1)	14 (4.7)
Sufentanil, µg^a^	90 (70–100)	90 (65–100)
High vasopressor support^b^	196 (83.4)	52 (81.3)
Duration of anesthesia, min	265 (235–290)	270 (237.5–302.5)
Fluids, ml^c^	2500 (2000–3000)	2500 (2000–3000)
PSA preoperative, ng/ml	7.2 (5.2–11.2)	7.7 (4.8–12.8)
Neoadjuvant androgen deprivation therapy	11 (4.7)	7 (10.9)
Prostate volume (sonographic assessment), ml	36 (28–50)	44 (29–56)
Prostate volume (histopathologic assessment), ml	25 (20–35)	30.1 (20–45)
Tumor volume, ml	4.6 (2.4–8.4)	5.4 (3.1–12.2)
Resected lymph nodes	14 (8–22)	13 (7–21)
*N stage*		
N0	187 (79.6)	42 (65.6)
N1	31 (13.2)	16 (25.0)
Nx	17 (7.2)	6 (9.4)
*T stage*		
pT2a-2c	155 (66.0)	32 (50.0)
pT3a-3c	80 (34.0)	32 (50.0)
*Gleason score*^d^		
1	15 (6.4)	4 (6.3)
2	144 (61.3)	33 (51.6)
3	52 (22.1)	15 (23.4)
4	2 (0.9)	0 (0.0)
5	22 (9.4)	12 (18.8)
*Positive surgical margin*		
R0	187 (79.6)	43 (67.2)
R1	48 (20.4)	20 (31.3)
Rx	0 (0.0)	1 (1.6)

*DNCR* delayed neurocognitive recovery, *RARP* robot-assisted radical prostatectomy, *ORP* open retropubic radical prostatectomy, *TIVA* total-intravenous anesthesia, *PSA* prostate-specific antigen

^a^Total dose administered for anesthesia induction and intraoperatively

^b^High vasopressor support was defined as requirement of noradrenaline > 75% of surgical time

^c^Total intraoperative amount

^d^Grading according to the 2014 International Society of Urological Pathology Consensus Conference

### Follow up 12 months after surgery

**Fig. 2:**
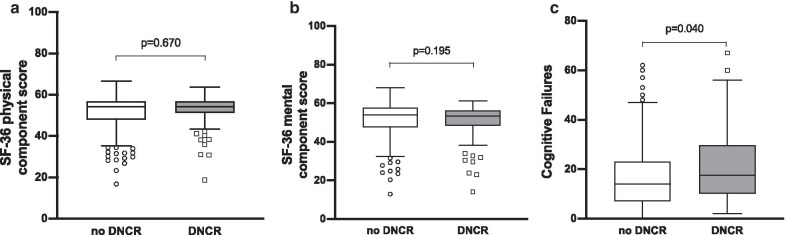
**a**–**c** Median with interquartile range of the physical (**a**) and mental (**b**) component scores of the Short Form Health Survey (SF-36) and self-reported cognitive failures (**c**) 12 months after radical prostatectomy. Scores were compared between patients without delayed neurocognitive recovery (DNCR) and with DNCR in the early postoperative period with the Mann–Whitney-U test

Physical HRQoL 12 months after surgery did not differ significantly between patients with and without DNCR in the early postoperative phase (54.2 [IQR: 51.4–56.8] vs. 54.3 [IQR: 48.0–56.9], *p* = 0.670; Fig. 2a). There was no significant difference in mental HRQoL 12 months after prostatectomy between patients with and without DNCR (53.5 [IQR: 48.4–56.2] vs. 54.0 [IQR: 47.6–57.8], *p* = 0.195; Fig. 2b). Cognitive failures were significantly higher in patients with DNCR than in patients without DNCR during the early postoperative period (17.5 [IQR: 10.0–29.5] vs. 14.0 [IQR: 7.0–23.0], *p* = 0.040; Fig. 2c). Details on SF-36 subscales and cognitive failures are given in Additional file 3.

The correlations between physical HRQoL and mental HRQoL (rs = 0.151; *p* = 0.009) and between physical HRQoL and CFQ score (rs =  − 0.247; *p* < 0.001) were negligible. We found a low negative correlation between mental HRQoL and CFQ score (rs =  − 0.427; *p* < 0.001).

### Delayed neurocognitive recovery and patient-reported outcome measures

Variable selection with categorical nonlinear principal components analysis is presented in Additional file 1. Delayed neurocognitive recovery in the early postoperative period and high preoperative depression screening scores were significantly associated with self-reported cognitive failures 12 months after surgery (Table 3). There was no significant association between DNCR and physical HRQoL at 12 months. High preoperative depression ratings, age, and tumor volume and neoadjuvant androgen deprivation therapy (ADT) were significantly associated with lower physical HRQoL at 12 months (Table 3). There was no significant association between DNCR and mental HRQoL 12 months after surgery. High depression screening scores at the preoperative baseline assessment were significantly associated with lower mental HRQoL (Table 3).

**Table 3:** Results of the general linear models for factors associated with self-reported cognitive failures and health-related quality of life (Short Form Health Survey SF-36) 12 months after radical prostatectomy

	B	95% CI	*p*
*Cognitive failures*			
No DNCR (vs. DNCR)	− 0.41	− 0.80; − 0.02	0.038
Depression (PHQ-9, per point increase)	0.13	0.09; 0.18	< 0.001
*SF-36 physical component score*			
No DNCR (vs. DNCR)	0.08	− 0.02; 0.19	0.118
Depression (PHQ9, per point increase)	0.02	0.01; 0.03	0.001
Age (per year increase)	0.01	0.01; 0.02	0.001
Tumor volume (per ml increase)	0.01	0.00; 0.01	0.005
No ADT (vs. ADT)	− 0.23	− 0.42; − 0.05	0.014
*SF-36 mental component score*			
No DNCR (vs. DNCR)	− 0.04	− 0.15; 0.06	0.417
ASA I (vs. ASA III)	− 0.03	− 0.18; 0.12	0.712
ASA II (vs. ASA III)	0.05	− 0.08; 0.17	0.477
Depression (PHQ-9, per point increase)	0.06	0.05; 0.07	< 0.001
Age (per year increase)	− 0.00	− 0.01; 0.00	0.464
Tumor volume (per ml increase)	0.01	0.00; 0.01	0.044

*DNCR* delayed neurocognitive recovery, *PHQ-9* patient health questionnaire, *ADT* neoadjuvant androgen deprivation therapy, *ASA* American Society of Anesthesiologists physical status classification

## Discussion

In our study, DNCR in the early postoperative period had no adverse effect on either physical or mental HRQoL 12 months after radical prostatectomy. However, DNCR before hospital discharge was associated with a higher incidence of self-reported cognitive failures one year after surgery. Furthermore, our results suggest that physical and mental HRQoL and cognitive failures at 12 months are associated with preoperative depressive symptoms. Higher age and tumor volume and treatment with ADT were associated with low physical HRQoL 12 months after surgery.

Interestingly, patients who met the criteria for DNCR in the early postoperative period reported more cognitive failures 12 months after surgery than patients without DNCR. This is in accordance with results from a previous observational study. Kastaun et al. compared preoperative and postoperative self-reported cognitive failures of patients with DNCR after cardiac surgery [20]. They found a deterioration of patient-reported cognitive function and cognitive performance as assessed by the patients’ next of kin from baseline to three months after surgery. One year postoperatively, cognitive function was still worse compared with baseline performance; however, these results were not statistically significant. Contrary to our study, Kastaun and colleagues did not compare CFQ scores of patients with and without DNCR, but rather assessed patients with postoperative cognitive decline only.

Results regarding the duration of postoperative neurocognitive disorders after non-cardiac surgery are conflicting. It is unclear whether cognitive impairment persists beyond three months since few studies have addressed the prevalence of postoperative neurocognitive disorders in the long term.

Some studies report that cognitive function at 12 months is similar to that among the general population [10, 12]. By contrast, an observational study found postoperative neurocognitive disorders in up to 76% of patients 12 months after non-cardiac surgery [11]. Our findings suggest that DNCR in the early postoperative period still affects memory, attention, action, and perception in daily life one year after radical prostatectomy. This underlines the long-term effects of DNCR and its potential adverse consequences in terms of individual and socioeconomic impact [7, 13].

Contrary to our hypothesis, we did not find an association between either the physical or mental components of HRQoL at one year and DNCR in the early postoperative period. Few studies have addressed the relationship between DNCR after surgery and long-term HRQoL. The influence of DNCR on HRQoL after cardiac surgery has been investigated in two longitudinal studies. Newman and colleagues report DNCR in 53% of study participants at discharge and postoperative neurocognitive disorders in 36% at six weeks and 24% at six months [21]. The same research group found a 41% incidence rate of postoperative neurocognitive disorders six weeks after surgery and 36.8% one year after surgery [22].

When comparing our results with the two aforementioned studies, it is important to note that the incidence and the type of DNCR may differ between cardiac and non-cardiac surgeries [23, 24]. The reason for this has been a matter of debate and may be attributable to diverse patient-related risk factors [24, 25]. The lack of association between DNCR following non-cardiac surgery and HRQoL after one year in our study might result from a lower severity or different type of postoperative cognitive decline compared with that experienced after cardiac surgery.

We found preoperative depression scores to be significantly associated with patient-reported outcomes. Both physical and mental HRQoL and cognitive failures 12 months after surgery were negatively affected by higher preoperative depression screening scores. Results from a longitudinal study suggest that depression has a negative impact on HRQoL in patients with prostate cancer [26]. This effect has also been reported in the perioperative context. Patients with elevated preoperative ratings for depression had significantly reduced HRQoL after general surgery for oncological disease [27]. Furthermore, depression not only has a negative impact on HRQoL but is also directly correlated with cognitive failures [28]. This is true for both major depressive disorder and seasonal depressive disorder, in which cognitive failures wax and wane with the severity of depressive symptoms [29]. These findings underline the importance of preoperative screening for depressive symptoms in patients with prostate cancer to allow for early psycho-oncological intervention.

The multivariable analysis showed that physical and mental HRQoL 12 months after surgery were negatively influenced by high tumor volume. Physical HRQoL was also associated with higher age and ADT. These findings are in accordance with results from a previous study. High tumor volume has been linked to cancer recurrence after radical prostatectomy for prostate cancer [30]. Cancer recurrence may entail the resurgence of clinical symptoms, additional treatment, or even hospitalization and, therefore, reduced physical HRQoL. Previous studies have found higher age to be related to poorer physical HRQoL in prostate cancer survivors, whereas younger patients have been found to experience a greater decline in specific uro-oncological aspects of HRQoL, such as sexual and urinary function [31]. Androgen deprivation therapy has various adverse effects, which may lead to reduced HRQoL, bone health, and cardiovascular health [32]. The main causes of ADT-related decreased HRQoL are treatment-induced loss of libido, erectile dysfunction, fatigue, cognitive impairment, depression, gynecomastia, and anemia [32].

This trial has several limitations that must be acknowledged. The study protocol was initially designed to detect a difference in the incidence of DNCR between robot-assisted radical prostatectomy and open retropubic surgery. Therefore, findings on HRQoL and cognitive failures are of an exploratory nature and should be interpreted with caution.

The SF-36 is one of the most commonly used questionnaires for HRQoL in patients with prostate cancer [33]. We chose to use a generic instrument over a prostate cancer-specific questionnaire, as our aim was to investigate the influence of DNCR on HRQoL rather than the impact of uro-oncological features on patient-reported outcomes in prostate cancer.

We did not assess cognitive failures and HRQoL preoperatively. Therefore, we cannot evaluate a potential change in HRQoL from preoperative to postoperative values. Importantly, the aim of this follow-up study was to assess the impact of DNCR after radical prostatectomy on long-term HRQoL rather than to analyze trajectories of perioperative HRQoL. We do not know whether patients who presented with DNCR in the early postoperative period might have experienced more cognitive failures preoperatively. However, we used the Mini-Mental Status Examination as an objective screening tool to detect preoperative cognitive impairment and found no differences between patients with and without DNCR.

Finally, we had information about neoadjuvant ADT but not about whether adjuvant ADT (+/− adjuvant radiation therapy) was administered. Both of these can impact postoperative HRQoL and are usually associated with a more advanced tumor stage [34]. Considering that we evaluated men 12 months post-surgery, the impact of adjuvant ADT, which is typically administered for 6–24 months, and its associated effects might be more profound than that of neoadjuvant ADT. Given the fact that neoadjuvant ADT had a significant impact, it is reasonable to assume that such an impact might be even more profound with adjuvant ADT.

One strength of this trial is the homogeneity of our study population with regard to gender, perioperative risk, and education. It is important to note, however, that our results may not be generalizable to patients who undergo non-cardiac surgery other than radical prostatectomy. For assessing HRQoL and cognitive failures 12 months after surgery, 20% of participants were lost to follow-up. Therefore, we cannot exclude a selection bias.

## Conclusions

Delayed neurocognitive recovery in the early postoperative period was associated with higher cognitive failures 12 months after surgery. Our findings suggest that DNCR has a long-term impact on patients’ daily lives by impairing memory, attention, action, and perception. Therefore, the prevention, recognition, and treatment of DNCR must be a priority for physicians and researchers. In patients who underwent robot-assisted or open radical prostatectomy, the presence of DNCR before hospital discharge had no adverse effect on either physical or mental HRQoL 12 months postoperatively. Higher preoperative depression ratings were associated with lower physical and mental HRQoL and with higher cognitive failures. Preoperative screening for depressive symptoms and consequent treatment are recommended to facilitate early intervention to improve postoperative HRQoL.

## Supplementary Information

**Additional file 1.** Nonlinear categorical principal components analysis (CATPCA) was used to graphically display the relationship between the initial set of variables [35, 36]. Clinically relevant variables subjected to CATPCA were: age, Body Mass Index (BMI), American Society of Anesthesiologists physical status (ASA), education, cardiovascular risk, pN stage, Gleason score, tumor volume, positive surgical margin, pT stage, prostate-specific antigen (PSA, preoperative), androgen deprivation therapy (ADT), estimated blood loss, type of surgery, colloid fluids, preoperative Patient Health Questionnaire-9 (PHQ9), preoperative Mini-Mental Status Examination (MMSE). Angles between vectors of variables indicate their degree of correlation (cosine of angle **≙** correlation); the vector lengths indicate the explanatory value of variables. For example, variables pN-stage and tumor volume are extremely highly positively correlated (collinear) but pN-stage has a somewhat lower explanatory value of the two. From among highly correlated variables, only those with the highest explanatory value and/or highest clinical relevance were considered in the general linear models: age, BMI, ASA, education, Gleason score, tumor volume, ADT, type of surgery, PHQ9, MMSE.

**Additional file 2.** Deterioration of postoperative cognitive function was based on the calculation of z-scores ([postoperative result—preoperative result]/SDpre-OP). Data are presented as median with interquartile range. DNCR = delayed neurocognitive recovery; CVLT = California verbal learning test.

**Additional file 3.** Cognitive failures assessed with the Cognitive Failures Questionnaire and health-related quality of life 12 months after radical prostatectomy. Health-related quality of life was evaluated with the Short-Form Health Survey SF-36. Eight subscales and two component summary scores are presented as medians with interquartile ranges.

## Data Availability

The datasets generated and/or analyzed during the current study are not publicly available due to the General Data Protection Regulation and the Federal Data Protection Act, but are available from the corresponding author on reasonable request.

## Abbreviations

ADT: Androgen deprivation therapy
CFQ: Cognitive failures questionnaire
DNCR: Delayed neurocognitive recovery
HRQoL: Health-related quality of life
IQR: Interquartile range
SF-36: Short Form Health Survey
