# Supplementary figures and images for: Health-related quality of life and self-reported cognitive function in patients with delayed neurocognitive recovery after radical prostatectomy: a prospective follow-up study
Source: Health Qual Life Outcomes. 2021 Feb 25;19:64. doi: 10.1186/s12955-021-01705-z (PMC7908756; doi:10.1186/s12955-021-01705-z)

# Component Loadings

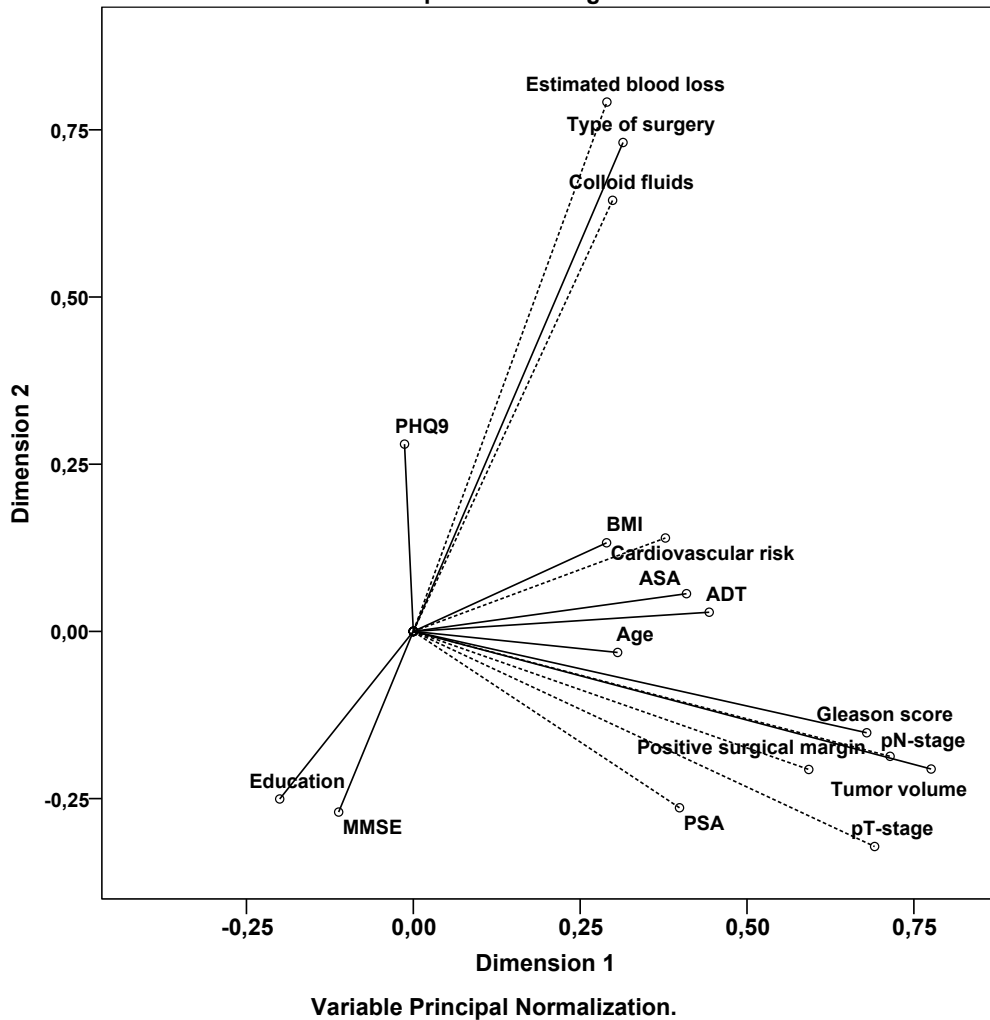

Supplement: Supplementary file 1 — Additional file 1. Nonlinear categorical principal components analysis (CATPCA) was used to graphically display the relationship between the initial set of variables [35, 36]. Clinically relevant variables subjected to CATPCA were: age, Body Mass Index (BMI), American Society of Anesthesiologists physical status (ASA), education, cardiovascular risk, pN stage, Gleason score, tumor volume, positive surgical margin, pT stage, prostate-specific antigen (PSA, preoperative), androgen deprivation therapy (ADT), estimated blood loss, type of surgery, colloid fluids, preoperative Patient Health Questionnaire-9 (PHQ9), preoperative Mini-Mental Status Examination (MMSE). Angles between vectors of variables indicate their degree of correlation (cosine of angle ≙ correlation); the vector lengths indicate the explanatory value of variables. For example, variables pN-stage and tumor volume are extremely highly positively correlated (collinear) but pN-stage has a somewhat lower explanatory value of the two. From among highly correlated variables, only those with the highest explanatory value and/or highest clinical relevance were considered in the general linear models: age, BMI, ASA, education, Gleason score, tumor volume, ADT, type of surgery, PHQ9, MMSE. [file 12955_2021_1705_MOESM1_ESM.pdf]
