# Supplementary material for: Health-related quality of life and self-reported cognitive function in patients with delayed neurocognitive recovery after radical prostatectomy: a prospective follow-up study
Source: Health Qual Life Outcomes. 2021 Feb 25;19:64. doi: 10.1186/s12955-021-01705-z (PMC7908756; doi:10.1186/s12955-021-01705-z)
Supplement: Supplementary file 2 — Additional file 2. Deterioration of postoperative cognitive function was based on the calculation of z-scores ([postoperative result—preoperative result]/SDpre-OP). Data are presented as median with interquartile range. DNCR = delayed neurocognitive recovery; CVLT = California verbal learning test. [file 12955_2021_1705_MOESM2_ESM.docx]

## Additional file 2

|  | no DNCR  (n=235) | DNCR  (n=64) |
| --- | --- | --- |
| Time point of neuropsychological assessment (postoperative day) | 3 (3-4) | 3 (2-4) |
| CVLT total recall | -0.2 (-0.8-0.3) | -0.41 (-1.01-0.2) |
| CVLT learning slope | -0.17 (-0.68-0.68) | -0.17 (-1.01-0.51) |
| CVLT vulnerability to proactive interference | -0.02 (-0.76-0.96) | 0.24 (-0.7-0.99) |
| CVLT vulnerability to retroactive interference | -0.27 (-0.83-0.51) | 0.1 (-0.94-1.42) |
| CVLT retention of information  over short intervals | -0.38 (-1.1-0.35) | -0.15 (-1.28-1.08) |
| CVLT retention of information  over longer intervals | -0.26 (-1.25-0.5) | -0.17 (-2.03-0.64) |
| CVLT intrusion error-types | 0.0 (-0.56-0.56) | 0.19 (-0.38-1.32) |
| CVLT discriminability | -0.47 (-1.2-0.03) | -1.39 (-2.14-(-)0.07) |
| CVLT encoding and retrieval | 0.01 (-0.78-0.92) | -0.38 (-1.95-1.08) |
| Trail making test - B | -0.03 (-0.39-0.4) | -0.06 (-0.46-0.81) |
| Grooved Pegboard (dominant hand) | 0.12 (-0.3-0.42) | 0.33 (-0.42-0.88) |
| Digit span forward | 0.0 (-0.98-0.0) | 0.0 (-0.98-0.98) |

Additional file 2: Deterioration of postoperative cognitive function was based on the calculation of z-scores ([postoperative result – preoperative result]/SD_pre-OP_). Data are presented as median with interquartile range. DNCR = delayed neurocognitive recovery; CVLT = California verbal learning test.
