# Supplementary material for: Health-related quality of life and self-reported cognitive function in patients with delayed neurocognitive recovery after radical prostatectomy: a prospective follow-up study
Source: Health Qual Life Outcomes. 2021 Feb 25;19:64. doi: 10.1186/s12955-021-01705-z (PMC7908756; doi:10.1186/s12955-021-01705-z)
Supplement: Supplementary file 3 — Additional file 3. Cognitive failures assessed with the Cognitive Failures Questionnaire and health-related quality of life 12 months after radical prostatectomy. Health-related quality of life was evaluated with the Short-Form Health Survey SF-36. Eight subscales and two component summary scores are presented as medians with interquartile ranges. [file 12955_2021_1705_MOESM3_ESM.docx]

## Additional file 3

|  | no DNCR  (n=235) | DNCR  (n=64) |
| --- | --- | --- |
| Cognitive failures | 14 (7-23) | 18 (10-30) |
| Physical functioning | 95 (85-100) | 95 (85-100) |
| Role-physical | 100 (75-100) | 100 (75-100) |
| Bodily pain | 100 (74-100) | 100 (84-100) |
| General health | 72 (57-82) | 67 (57-82) |
| Vitality | 65 (55-80) | 65 (55-75) |
| Social functioning | 100 (75-100) | 100 (75-100) |
| Role-emotional | 100 (100-100) | 100 (67-100) |
| Mental health | 80 (68-88) | 82 (72-88) |
| Physical component summary | 54.3 (48.0-56.9) | 54.2 (51.4-56.8) |
| Health component summary | 54.0 (47.6-57.8) | 53.5 (48.4-56.2) |

Additional file 3: Cognitive failures assessed with the Cognitive Failures Questionnaire and health-related quality of life 12 months after radical prostatectomy. Health-related quality of life was evaluated with the Short-Form Health Survey SF-36. Eight subscales and two component summary scores are presented as medians with interquartile ranges.
